# Deep Learning Techniques for Vehicle Detection and Classification from Images/Videos: A Survey

**DOI:** 10.3390/s23104832

**Published:** 2023-05-17

**Authors:** Michael Abebe Berwo, Asad Khan, Yong Fang, Hamza Fahim, Shumaila Javaid, Jabar Mahmood, Zain Ul Abideen, Syam M.S.

**Affiliations:** 1 School of Information and Engineering, Chang’an University, Xi’an 710064, China; 2019024902@chd.edu.cn (M.A.B.); fy@chd.edu.cn (Y.F.); 2019024906@chd.edu.cn (J.M.); 2 School of Computer Science and Cyber Engineering, Guangzhou University, Guangzhou 510006, China; 3 School of Electronics and Information, Tongji University, Shanghai 200070, China; shumaila@tongji.edu.cn; 4 Research Institute of Automotive Engineering, Jiangsu University, Zhenjiang 212013, China; 1000006198@ujs.edu.cn; 5 IOT Research Center, Shenzhen University, Shenzhen 518060, China; syamms@email.szu.edu.cn

**Keywords:** deep learning, vehicle detection and classification, CNN, activation function, loss function

## Abstract

Detecting and classifying vehicles as objects from images and videos is challenging in appearance-based representation, yet plays a significant role in the substantial real-time applications of Intelligent Transportation Systems (ITSs). The rapid development of Deep Learning (DL) has resulted in the computer-vision community demanding efficient, robust, and outstanding services to be built in various fields. This paper covers a wide range of vehicle detection and classification approaches and the application of these in estimating traffic density, real-time targets, toll management and other areas using DL architectures. Moreover, the paper also presents a detailed analysis of DL techniques, benchmark datasets, and preliminaries. A survey of some vital detection and classification applications, namely, vehicle detection and classification and performance, is conducted, with a detailed investigation of the challenges faced. The paper also addresses the promising technological advancements of the last few years.

## 1. Introduction

Object detection and classification have received a lot of attention in recent years due to the wide range of applications that are possible and the recent flurry of activity in computer vision research. Most applications in ITS regarding vehicle detection and classification focus a great deal of effort on traffic accident investigation, traffic flow monitoring, fleet and transport management, autonomous driving, and similar. Digital image processing techniques have been aggressively employed in recent years in vehicle shape detection, color, speed, and post estimation. Simultaneously, computational power has increased. Nowadays, computer vision-based [1,2,3] platforms are equipped with high-core processing and graphics processing units (GPUs), which detect and classify objects to pursue real-time implementations. Deep Learning (DL) and Machine Learning (ML) have exhibited vital CV research applications. Deep ConvNets have various architectures of DL on CV topics, such as image classification, object detection, object recognition, learning, vehicle tracking, object pose estimation, and others.

An image is a two-dimensional digital distribution of pixel values designated by finite numbers. The pixels are denoted on the x–y spatial coordinate axis [4]. Digital image processing is a term that describes the processing of an image or video frame, taken as input, and involving a set of phases with various parameters and experimental setups. For example, detecting a vehicle would imply that images or video frames clearly show its presence, as well as its location, in an image. Therefore, object detection can be defined as a means to locate samples of real-world objects in images. In this context, vehicle detection is closely related to vehicle classification, since it involves defining the presence and location of the vehicle in an image. However, the image is useless unless it is properly analyzed to extract useful knowledge. Hand-crafted features (namely, Histogram of Oriented Gradient (HOG) [5], Haar [6], and LBP [7]) are the most appropriate techniques to detect vehicles, but they fail to provide a general solution, and the classifiers require some modifications to fit various parameters. A shallow neural network is utilized as well for vehicle detection, though its performance has not provided the desired quality. Handling this massive amount of data necessitates the growth of an innovative method capable of performing quickly, precisely, and consistently. Advancing the efficiency of vehicle detection and classification accuracy, precision, and robustness through DL techniques, such as DCNNs, RCNNs, and DNNs, improves the robustness of schemes in detecting and classifying vehicles from images or video frames.

Rapid improvement and innovative ideas are utilized to improve the accuracy of detection and classification of DL schemes and to reduce computational costs during the training and testing phases of DL schemes. Among these innovative approaches are those involving the modification of DCNNs, transferring learning (TL), hyper-parameter optimization, and implementation of image-preprocessing techniques (enhancement, scaling, median filtering, fuzzy filtering, and Ensemble Learning (EL), in the proposed DL architectures. For better understanding, the abbreviations are given in the Abbreviations section.

**Figure 1:**
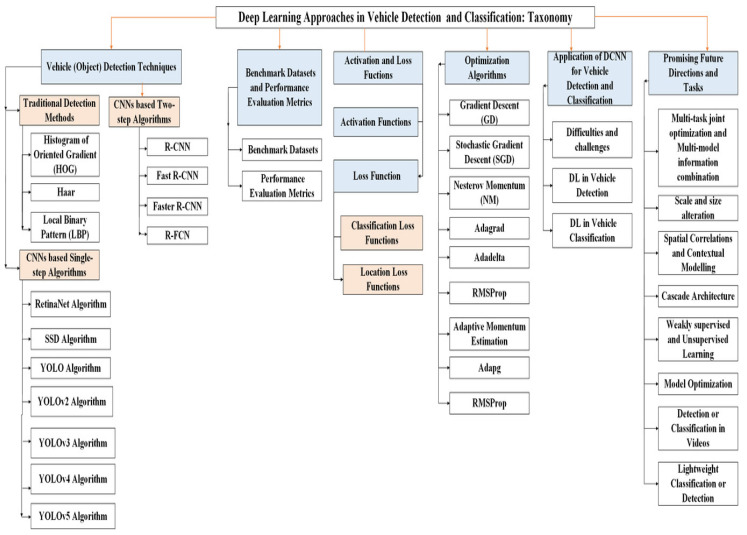
Taxonomy of the Deep Learning Approaches in Vehicle Detection and Classification Tasks.

The main contributions of this survey article are as follow:

- We survey the methodologies, benchmark datasets, loss and activation functions, and optimization algorithms used in vehicle identification and classification in deep learning.
- We survey the strategies for vehicle detection and classification studies in Deep Convolutional Neural Networks.
- We address the taxonomy of deep learning approaches and other functions in object detection and classification tasks (as shown in Figure 1).
- We present promising technological future directions and tasks in improving deep learning schemes for researchers.

This paper is organized into the following sections. Section 2 explains a detailed analysis of DL techniques. Section 3 discusses the publicly available benchmark datasets and performance evaluation metrics. Section 4 explains the application of activation and loss functions in DL. Section 5 explains the optimization algorithms in DL. Section 6 explains applications of DL in vehicle detection and classification and compares recently employed techniques. Section 7 briefly discusses some promising future directions and tasks that have been adopted to improve and optimize DL schemes and to solve the difficulties and challenges that occur during training and testing of the models. Section 8 is the conclusion of the survey.

## 2. Deep Learning Techniques

Object detection, recognition, and classification in computer vision are practically helpful but technologically challenging. There are two main categories: multi-oriented object detection and classification and single object recognition. DL approaches for object detection and recognition and classification of images mainly focus on accurate object recognition (improving detection and recognition performance), speed of testing, training, computational processes, and accurate object classification (minimizing the error rate) [8,9].

Deep Learning deals with DNN architecture, where deep refers to figures of the hidden layers, and its main objective is to resolve learning problems by copying the functioning of the human brain [9,10]. Schemes employing DL have been developing and improving consistently, as have adjustments to the model structure. Depending on the scheme, tuning may be required or setups applied to upgrade the execution of the scheme. The designs of DCNNs often involve the following essential elements:

Convolution Layer: The convolution layer is the initial layer that receives an input image and extracts the features from that data. It utilizes small input data and learns the data features by sustaining the correlation between values of pixels, which involves a filter/kernel matrix and an image matrix, and the performance of a mathematical operation to learn the features.

Activation Function: Linear or non-linear activation functions are used to monitor the results of models. They can be linear or non-linear, depending on the function they monitor.

Pooling Layers: These employ subsampling and spatial pooling techniques to minimize some parameters without removing the critical parameter. Various methods of pooling are employed, including average, sum, and maximum approaches.

**Figure 2:**
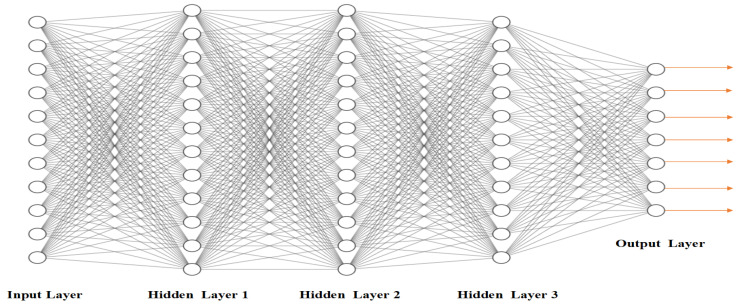
A Deep Nets Architecture.

Fully Connected (FC) Layer: The final few layers are FC layers. After the final pooling or CNN layer, the output feature maps are mainly flattened (vectors) and used as input to FC layers. A Deep Nets Architecture is depicted in Figure 2.

### 2.1. Techniques

In this subsection we discuss deep learning techniques.

#### 2.1.1. Traditional Detection Methods

**Figure 3:**
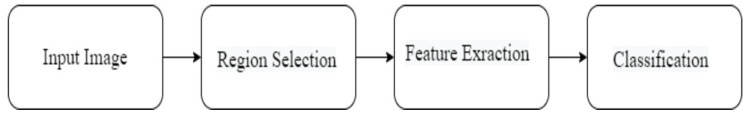
Traditional Feature-based object Recognition and Classification Architecture.

In more recent years, object recognition/detection and classification have been hot research topics in computer vision-based applications. Various objects in various environments may be challenging to detect, and, therefore, to classify and identify, due to the following factors: weather, lighting, illumination effects, size of the objects, inter-class variations, intra-class variations, and other factors. In recent studies, many extracted AI features have been employed to classify objects. The traditional feature-based object recognition and classification approaches consist of three systems (see Figure 3):

- Region selection
- Feature extraction, and
- Classification.

The most common traditional feature-based architectures in the literature for vehicle detection and recognition and classification are the Histogram of Oriented Gradient (HOG) [5], Haar [6], and LBP [7].

Haar features are calculated by adding and subtracting the sums of rectangles and the differences across an image patch. As this was highly efficient at calculating the symmetry structure in detecting vehicles [11], it was ideal for real-time detection. The Haar feature vector and the AdaBoost [12,13] were widely used in CV to detect objects in a variety of feature applications, including vehicle recognition [11].

HOG features are extracted in the following phases:

- Evaluating the edge and discretizing the image;
- Removing edge sharpness.

The HOG feature vector integrated with the Support Vector Machine (SVM) classifier has been widely employed to recognize object orientation, i.e., on-road vehicle detection [14,15]. The HOG–SVM [16] performed admirably in multi-vehicle detection tasks. In addition, a blend of HOG [5] and Haar [6] was employed for vehicle recognition, detection, and tracking [17].

Local Binary Pattern (LBP) [7] features have performed better in different applications, including texture classification, face recognition, segmentation, image retrieval, and surface crack detection. The cascade classifier (Haar–LBP–HOG feature) [18] is detects vehicles with bounding boxes. In addition to the previously mentioned features and classifiers for vehicle detection and classification problems, statistical architectures, based on horizontal and vertical edge features, were proposed for vehicle detection [19], side-view car detection [20], online vehicle detection [21], and vehicle detection in severe weather using HOG–LBP fusion [22].

#### 2.1.2. CNN-Based Two-Step Algorithms

A two-step object detector, or the region-based approach, comprises two steps to process an image:

- Produce a series of candidate frames or extract region proposals from the scene;
- Classify and regress the generated candidate frames to improve the architecture’s detection accuracy.

**Figure 4:**
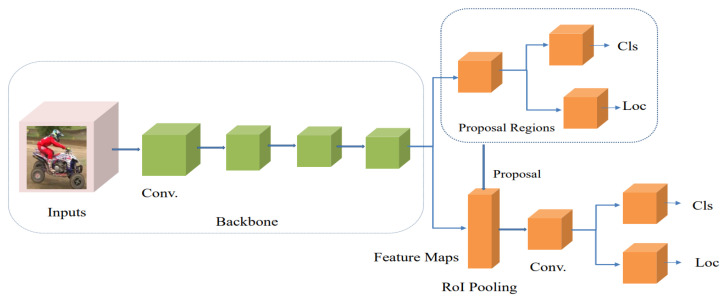
Basic Architecture of Two-step Detector.

The region-based approach has the properties of high localization and performance, slower speed, and high computational cost during training. Figure 4 displays the architecture of a two-step object detector. Researchers have proposed several two-step object detector algorithms and these have been employed for vehicle detection and classification in more recent years. They are explained as follows:

R-CNN: Girshick et al. [23] proposed an R-CNN or region-based ConvNet two-step object detector architecture. In [23,24] AlexNet was employed as the backbone model of the detector. It can increase the detection accuracy of objects over that of traditional object detection algorithms, such as HOG [5], Haar [6] and LBP [7] feature extraction. The R-CNN has four systems to accomplish the tasks. The operation of the algorithm is as follows:

- Produce categorical-independent region proposals;
- Extract a fixed-length feature vector from each region proposal;
- Compute the confidence scores to classify the object classes using class-specific support vector machines;
- Predict the bounding-box regressor for accurate bounding-box predictions, once the object class has been classified.

The authors adopted a selective search approach [25] to search for parts of the image having higher probability. Convolutional neural networks (ConvNets) were used to extract a 4096 dimensional feature vector from each proposed region. There had to be an exact match in length between the region’s proposed features and the input vectors for the FC. For the model, the authors used a fixed pixel size of 27×27, regardless of the candidate region’s size or aspect ratio. When using R-CNN, the final FC is linked to the M+1 classification layers (hence, *M* represents the number of object classes and 1 represents the background) to perform the final object classification. Optimizing convolution parameters, such as IoU, is accomplished with SGD. An IoU of less than 0.5 is considered incorrect for a region proposal; otherwise, it is correct. In R-CNN, without sharing computation, the region proposal and classification problems are carried out independently. However, R-CNN has problems concerning computational cost and training time for classification. To solve the problem of too much time required in the training process, convolutional feature maps with high resolution can be generated at a low cost using the Fast R-CNN architecture proposed by Girshick [26].

Fast R-CNN: The Fast R-CNN [26] network takes as input an entire image and a set of object proposals. It follows the following specific steps:

- Generate a convolution feature by using various convolution and max-pooling layers on the entire image;
- Extract a fixed-length feature vector from the feature map for each object proposal of Region of Interest pooling layers;
- Feed each feature vector into a sequence of FC layers to generate softmax probability predictions over *M* object classes plus 1 background (M+1). The other layer generates four real-valued *n*. Fast R-CNN utilizes a streamlined training process with a fine-tuning step that jointly optimizes a softmax classifier and Bbox regressors.

Training a softmax classifier, SVMs, and regressors in separate stages accelerates the training time over the standard R-CNN architecture. The entire process architecture includes loss, the SGD optimizer, the mini-batch sampling strategy, and BP through the RoI pooling layers. However, Fast R-CNN uses a selective search approach over the convolution feature map to explore its pooling map, increasing its run time. Using a new region proposal network (RPN), Shaoqing et al. [27] proposed a faster RCNN architecture to improve the Fast RCNN network in terms of run time and detection performance in order to better estimate the object region at various aspect ratios and scales.

Faster R-CNN: In terms of operation time and detection performance, the faster RCNN [27] is a more advanced variant of the RCNN. Instead of the traditional method, selective search replaces RPN’s outstanding prediction of object regions at various scales and aspect ratios. Anchors are placed at each convolutional feature location to create a variety of region proposals. The anchor box in Faster RCNN has three different aspect ratios and three different scales.

It comprises four systems to achieve object detection tasks: candidate region producing, feature extraction, classification, and location fine-tuning. In the RPN architecture, the feature map is computed using a sliding window of 3×3, which is then output to the Bbox classification and Bbox regression layers. Each point on the feature map is traversed by the sliding window, which places *z* anchor boxes where they are needed. The feature map’s *z* anchor boxes are used to extract its elements.

R-FCN: The two-step object detection architecture can be categorized into two distinct groups. One group represents classification networks like GoogleNet [28], ResNet [29], AlexNet [24], VGGNet [30]. Their computation is shared by all ROIs and an image test is conducted using one forward computation. In the second group, no computation is shared to all ROIs since it aims to classify the object regions. Dai et al. [31] proposed the R-FCN architecture of an improved version of the faster RCNN and partially eliminated the problem of position sensitivity and position variance by increasing the sharing of convolutional parameters. For the RFCN algorithm, the primary goal is the creation of “position-sensitive score maps.” If the ROI is not part of the object, it is determined by comparing it to the ROI sub-region, which consists of the corresponding parts (s×s). There is a shared convolutional layer at the end of the RFCN network’s network.

An additional layer of dimensional convolution ($4\times s^{2}$) is applied to the score maps to produce class-independent Bboxes. A softmax is used to calculate the results, after averaging the $s^{2}$ scores, to produce (M+1) dimensional vectors.

A comparison study was carried out on the most widely utilized two-step object detectors on both the COCO dataset [32] and the PASCAL VOC 07 [33] dataset. In [34], experimentation showed that RCNN achieved 66% of the mAP on the PASCAL VOC 07 dataset [33], while Fast RCNN achieved 66% of the same dataset. In addition, the Fast RCNN network was nine times faster than the standard RCNN network. Wang et al. [35] conducted a comparative study on three networks, namely, fast RCNN, faster RCNN, and the RFCN, on two publicly available datasets, i.e., the COCO [32] dataset and the PASCAL VOC 07 [33] dataset. On the COCO test dataset, faster RCNN improved detection accuracy by 3.2% compared to slow RCNN. Furthermore, the tasking positions on both RFCN and the faster RCNN on both datasets were compared. The experimental results revealed that RFCN outperformed the faster RCNN with superior detection accuracy and less operational run time. Table 1 displays the fundamental advantages and disadvantages of the most widely utilized two-step object detectors.

#### 2.1.3. CNN-Based Single-Step Algorithms

**Figure 5:**
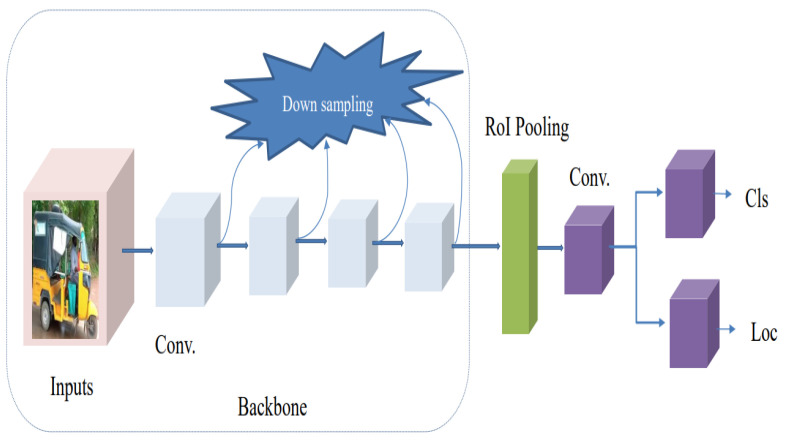
Basic Architecture of One-step Detector.

There is no region proposal phase for the classification or detection of object classes in a single-step algorithm, and the prediction results are directly obtained from the image. In this algorithm, the input image is sampled at various positions uniformly, using different aspect ratios and scales, and then the CNN layer is sampled to extract features to precisely execute regression and classification. The most notable merits of the models are that they are easier to optimize, suitable for real-time applications, and faster. There is no region proposal phase for the classification or detection of object classes in a single-step algorithm, and the prediction results are directly obtained from the image. In this algorithm, the input image uses a variety of aspect ratios and scales, and the CNN layer is sampled to extract features that can be used to accurately perform regression and classification. The most notable merits of the models are that they are easier to optimize, suitable for real-time applications, and faster. Figure 5 displays the framework of the Basic Architecture of One-step Detector. Numerous single-step object detector algorithms have been utilized for various applications, such as, among others, real-time vehicle object detection, vehicle recognition, in the last couple of years. Some of the most widely employed algorithms are the following: SSD [36], RetinaNet [37], YOLO [38], YOLOv2 [39], YOLOv3 [40], YOLOv4 [41], and YOLOv5 [42].

RetinaNet Algorithm: Lin et al. [37] proposed a RetinaNet algorithm that performs the focal loss as a classification loss. It solves the class imbalance between the positive and negative samples, which minimizes the prediction accuracy. The author introduced a focal loss to minimize the weight loss by avoiding several negative samples given in the background. The algorithm utilizes the ResNet [43] model as a backbone and FPN [44] as feature extraction architecture. It consists of two processes: generating a set of region proposals via FPN and classification of each candidate.

SSD Algorithm: Liu et al. [36] proposed an SSD algorithm based on a feedforward convolutional architecture that generates a fixed-size sum of bounding boxes and scores for existing object class samples, followed by an NMS stage to generate the detection process. The SSD algorithm utilizes a VGG16 [43] architecture as a backbone for feature extraction and six more convolutional layers for detection. It generates sequences of feature maps of various scales, followed by a 3×3 filter on each feature map to generate default Bboxes. It only detects at the top layers to get the best prediction Bbox and class label.

YOLO Algorithm: The YOLO algorithm [38] is a CNN-based object detection one-step detector that was designed after two-step object detection became the faster RCNN detector. The YOLO algorithm is most applicable for real-time image detection. It has a few region proposals per image compared to the faster RCNN. It utilizes a grid size of (t×t) to split the images into grid features for image classification. Grid cells can be used to estimate Bbox bounding boxes and *C* class probabilities for *C* object classes for each box. For each box, the probability (*P*) and the IOU between the ground truth and the box are considered. The YOLO algorithm has 2 FC layers and 24 convolution layers. However, the algorithm has the problem of weak object localization, which affects the classification accuracy.

YOLOv2 Algorithm: The YOLOv2 algorithm [39] is an improved version of the YOLO algorithm in detection precision and offers higher speed than the standard YOLO algorithm. It contains 6 consecutive tasks to efficiently perform the detection process, namely the BN, high-resolution classifier, convolution with anchor box, various aspect ratios and scales of the anchor box, fine-grained feature techniques, and multi-scale training.

The training process of the YOLOv2 algorithm [39] is carried out through the SGD optimizer, which employs a mini-batch. For example, mean, mini-batch, and variance are calculated and utilized for activation purposes.

Then, every mini-batch activation is normalized using the standard deviation of 1 and 0 mean. In the end, all elements in every mini-batch are sampled using an uniform distribution. This process is carried out through techniques of batch normalization (BN) [45]. It generates activation of uniform distribution to speed up its operation to obtain convergence. The YOLOv2 model uses a high-resolution classifier as a backbone to maximize the input resolution into (448×448), and classification fine-tuning is implemented for image resolution with 10 epochs to improve its map by 4%.

Moreover, techniques of convolution anchor box are also utilized to generate region proposals to predict the object-class score and class for each estimated Bbox, leading to an improvement of its recall by 7%. Furthermore, the model uses the anchor box’s size and aspect ratio prediction technique with *K*-means clustering. Fine-grained features for small objects and multi-scale training with image sizes of 320,352,...,608 improve the detection of objects of different sizes.

YOLOv3 Algorithm: The YOLOv3 Algorithm [40] is another improved version of the YOLO Algorithm. It utilizes the DarkNet53 model for feature extraction and employs a multi-label classification with overlapping patterns for the training process. It is primarily notable for object detection in complex scenes. In addition, in the YOLOv3 Algorithm, various sizes of three feature maps are utilized to predict the Bbox. The last convolution layer is used to produce a three-dimensional tensor that consists of objectness, class predictions, and Bbox.

YOLOv4 Algorithm: Single-step object detection algorithms, such as the YOLOv4 Algorithm [41], combine the properties of YOLO, YOLOv2, and YOLOv3 and achieve the current optimum in terms of both accuracy and speed. The residual system receives the feature layer and outputs the higher-level feature information. Algorithms like YOLOv4 are composed of a 3 structure called the “Neck”, “Backbone”, and “Prediction” sections. The SPPNet and PANet form the neck. Features in the SPPNet are concatenated and then extremely pooled by supreme cores of various scales in the feature layer. To increase the receptive field of the architecture, the pooled result is appended and convolved 3 times and the concatenated feature layers are up-sampled after concatenating with the SPPNet and Backbone. The process was cycled to up-sample and down-sample with feature layers to achieve CSPDarkNet53 for feature fusion and compression of height and width. Then, they are layered on top of each other to create new combinations of features. The features extracted from the model can be used to make predictions according to the prediction scheme. Prediction results from a network are filtered out using the Non-maximal Suppression (NMS) [46] efficient technique.

YOLOv5 Algorithm: The YOLOv5 algorithm utilizes CSPDarkNet as a backbone for the feature extraction model to extract feature information from the input data. Compared to the other variants of the YOLO algorithm, it has better capability to detect small objects, excellent detection accuracy, and is more adaptable and faster. It has 4 modules. The CSPNet architecture eliminates the gradient information duplication problem of model optimization in massive models and combines the gradient variation from the previous to the final into feature maps. Consequently, decreasing the volume of architecture FLOPS values and parameters causes the improved accuracy and speed of the model. However, it decreases the size of the architecture. The detection efficiency depends on the computation of the frame selection area to improve the model, which proposes the Fcos approach [47].

The model employs the CSPDarkNet feature extraction model to extract image features competently and utilizes Bottleneck CSP instead of a residual shortcut link to strengthen the description of the image features. The neck system is mainly employed to produce a feature pyramid. The feature pyramids can help the network find objects of different sizes, so as to find the frame object of different scales and sizes.

The CNN-based object detector has been applied to many DL-based applications. Its purpose is commonly illustrated as an effective, efficient object detection, recognition, and classification application with fewer error rates. The detector has been applied to face mask recognition [48,49], real-time vehicle detection [50], vehicle classification [51], off-road quad-bike detection [52], pedestrian detection [53], medical image classification [54], automotive engine crack detection [55] and so on.

Recent studies show that the CNN-based object detection algorithms (single-step and two-step object detectors) are gaining momentum in vehicle detection/recognition and classification. The algorithms are employed to detect and classify object classes from images and videos. Kausa et al. [56] utilized both single and two-step object detector approaches for two-wheeled and four-wheeled vehicle detection from publicly available datasets. Vasavi et al. [57] also applied integrated YOLO and RCNN algorithms for vehicle detection and classification from high-resolution images. In YOLOv3, a faster RCNN algorithm for detecting vehicles at night, using tail light images, was implemented, by [58].

It is essential to understand some of the object detection algorithms’ strengths and limitations (see Table 1 and Table 2). The detection and classification performance of the model is affected by various factors. Many studies have aimed to fix or decrease errors in predicting the exact object class and to ensure the algorithms work better.

We summarized the performance of the one-step and two-step object detectors on the COCO dataset and PASCAL VOC. The performance of deep learning-based object detection is affected by a series of elements, such as the following: feature extraction classifiers, type of backbone, image size and scale, training strategy, loss function, activation function, number of region proposals, etc. These elements make it challenging to compare several algorithms without a shared benchmark background. Table 3 shows the performance of the various algorithms employed in object detection tasks. The algorithms were compared using various performance evaluation metrics, such as FPs and average precision (AP) at inference time. The $AP_{0.5}$ represents the average precision of the object classes when the estimated Bbox has IoU>0.5 with ground truth and the $AP_{0.5-0.95}$ in 0.5 steps. The performances of the selected models were assessed on the same-sized input, where possible, to offer flexibility between inference time and detection accuracy.

## 3. Benchmark Datasets and Performance Evaluation Metrics

In this section, we describe the different benchmark datasets and performance evaluation metrics.

### 3.1. Benchmark Datasets

This section provides an overview of the common publicly available vehicle datasets utilized in vehicle detection, classification, and recognition tasks. Creating a large dataset volume under different lighting and weather conditions is challenging in vision-based architectures. The most famous vehicle datasets and benchmarks have been available for the last ten years, including the BIT vehicle dataset, comprehensive car datasets, KITTI benchmark datasets, Stanford car dataset, Tsinghua-Tencent Traffic Sign dataset, MotorBike7500, Tsinghua-Daimler Cyclist benchmark, etc.

BIT Vehicle Dataset: The challenge with the BIT Vehicle Dataset is the time-consuming effort required to speed up the growth of intelligent transportation system (ITS) vehicle type classification (VTC). In appearance-based tasks, it has been utilized in several applications, such as speed estimation, illegal vehicle detection, traffic flow, fleet management, and incident detection. It contains six object classes for every 150 vehicle to provide 900 vehicles: buses, microbuses, minibuses, SUVs, sedans, and trucks. Various conditions of illumination, time, color, viewpoint, and scale are applied. It introduced a classification accuracy of 93.8% and assessed the performance of the proposed model with an unlabeled vehicle over random values to capture rich discriminative information about vehicles for VTC.

CompCars Dataset: The Comprehensive Cars Dataset is one of the publicly available datasets. Images of both web and surveillance nature are included in the data set. It was launched in 2015, and its popularity has improved in the real-world application of appearance-based tasks. The web-nature scenario consists of 136,727 images that capture the entire part of the car and 27,618 car parts with labels and viewpoint. At the same time, the surveillance-nature data contains 44,481 images captured from the front view and annotated with Bbox, model, and color. The CompCar Dataset introduces four unique features compared to the other currently available datasets, such as car hierarchy, viewpoint, car attributes, and car parts.

KITTI Benchmark Dataset: The KITTI Benchmark Dataset [59,60] is one of the most widespread datasets used in autonomous traffic scenarios, consisting of various modalities, namely, high-resolution RGB, 3D laser scanner, and grayscale stereo cameras. Despite its popularity, the dataset does not have ground truth for segmentation purposes. However, many researchers have manually labeled the images to fit their needs for experimentation. Alvarez et al. [61,62] provided the ground truth of the dataset for 323 images from road detection challenges with three object classes: road, sky, and vertical. Further, Zhang et al. [63] labeled 252 captured RGB images from Velodyne scans and the tracking challenges for ten object classes: sky, car, building, vegetable, fence, cyclist, sidewalk, road, pedestrian, and sign pole. Ros et al. [64] also labeled 216 images from two odometer challenges from eleven object classes: sky, car, road, fence, bicyclist, sign, building, sidewalk, pedestrian, pole, and tree.

Stanford Car Dataset: The Stanford Car Dataset [65] is one of the publicly available car datasets for extensive research purposes. It contains 8144 training sample images and 8041 unseen images with object classes of 196 car types. It was launched in 2013, and its publicity has increased in object class detection and scene. Authors has extensively researched 3D object representations outperforming their 2D counter-parts for fine-grained categorization, and illustrated their effectiveness for estimating 3D geometry from images.

MotorBike7500 Dataset: The MotorBike7500 Dataset [66] is one of the benchmark motorcycle image datasets. It contains 7500 annotated images captured under real-time road traffic scenes with 60% occlusion rate. The images were resized to 640×364 pixels with 41,040 region of interest-annotated objects. The ground truth describes the frames covered by the objects, class, name, height, and width of the Bbox surrounding the object and provides an Id, which introduces a performance of 92% of the schemes on the benchmark dataset.

MotorBike10000 Dataset: The MotorBike10000 Dataset [66] is the extension of MotorBike7500 benchmark motorcycle image dataset. It contains a range of 10,000 annotated images captured under windy conditions with 60% occlusion rate. The images were resized to 640×364 pixels with 56,975 RoI annotated objects. The ground truth produced describes the frames covered by the objects, class, name, height, and width of the Bbox surrounding the object and provides an Id, which introduces the performance of 92% of the schemes on the benchmark dataset.

Tsinghua–Tencent Traffic Sign Dataset: The Tsinghua–Tencent Traffic Sign (TTTS) Dataset [67] consists of 30,000 samples of traffic signs and 100,000 images. The pictures are captured under diverse climatic conditions and lighting.

Tsinghua–Daimler Cyclist Benchmark: The Tsinghua–Daimler Cyclist Benchmark (TDCB) [68] provides a benchmark dataset for cyclist detection with six object classes: Mopedrider, pedestrian, Tricyclist, Cyclist, Wheelchair user, and Motorcyclist. It consists of Bbox of training, testing, and validation datasets of 16,202, 13,163, and 3045, respectively. Experimental results show an average precision of 89% for the easy case, which gradually reduces when the difficulty increases.

Cityscapes Dataset: The Cityscapes dataset [69] includes several collections of street scenes 20,000 and 500 weakly marked and full-length pictures from 50 different cities under diverse seasons, respectively.

GRAM Road-Traffic Monitoring (GRAM–RTM) Dataset: The GRAM–RTM Dataset [70] consists of video clips recorded under diverse conditions and on several platforms using surveillance cameras. It is widely utilized to evaluate the architecture of tracking several vehicles labeled in different classes, such as large trucks, cars, trucks, and vans. Each video clip contains 240 diverse object classes.

MIO–TCD Dataset: The MIO–TCD Dataset [71] is a dataset widely utilized for motorized traffic analysis. It consists of 11 object categories, such as motorcycles, bicycles, pedestrians, cars, buses, and trucks, with 786,702 labeled images captured under various times, seasons, and periods using traffic surveillance cameras.

UA–DETRACT Benchmark Dataset: The UA–DETRACT Benchmark Dataset [72] contains 100 video clips recorded at 24 diverse locations with diverse traffic patterns and conditions, such as traffic crossings, highways, and T-junctions, using a Canon EOS 550D camera.

LSVH Dataset: The LSVH Benchmark Dataset [73] consists of 16 video clips of vehicles with large-scale variations captured using surveillance cameras under diverse weather, scene, time, and resolution conditions.

COCO Dataset: The Microsoft COCO Benchmark Dataset [32] consists of 91 object classes of 328,000 images with 2,500,000 labeled samples. It is also significantly more prominent in several samples per class than PASCAL VOC [33].

PASCAL VOC Dataset: The PASCAL VOC Benchmark Dataset [33] is a publicly available dataset that contains annotated images collected from the Flickr photo-sharing website. It is a widely utilized dataset in object detection and classification to evaluate architectures.

ImageNet Dataset: The ImageNet Benchmark Dataset [74] consists of 80,000 synets of WillNet with an average of 500–1000 clean and full resolution images, having 12 subtrees with 5247 synets and 3.2 million images.

Caltech101 Dataset: The Caltech101 Benchmark Dataset [75] consists of images of 101 object classes. It is widely utilized in object recognition tasks.

Caltech256 Dataset: The Caltech256 Benchmark Dataset [76] is a series of the Caltech101 benchmark dataset which maximizes the object classes into 256 to improve the performance of multi-class object recognition with few training samples.

DAWN Dataset: The purpose of the DAWN Dataset [77] is to explore the effectiveness of vehicle detection and classification approaches of a wide range of natural images for traffic situations in the cross-generalization of adverse environmental conditions. It shifts substantially in terms of vehicle category, size, orientation, pose, illumination, position, and occlusion. Furthermore, this dataset demonstrates a systematic preference for traffic scenes during bad winter weather, heavy snowfall, sleet rain, hazardous weather, sand and dust storms.

### 3.2. Performance Evaluation Metrics

Object detectors and classifiers use several performance measures to quantify the performance of detectors and classifiers, namely, Precision (P), Frame per Second (FPS), Recall (R), True Positive Rate (TPR), False Positive Rate (FPR), Average mean Precision (AmP), intersection over union (IoU), average precision (AP), Accuracy, F1-Score, and Area Under Curve (AUC). The existing vehicle detection and classification approaches, as well as their corresponding performance measures, are shown in Table 4. In Table 5 demonstrates the various types of performance evaluation metrics and their mathematical equations.

## 4. Activation Functions in Deep Learning

**Figure 6:**
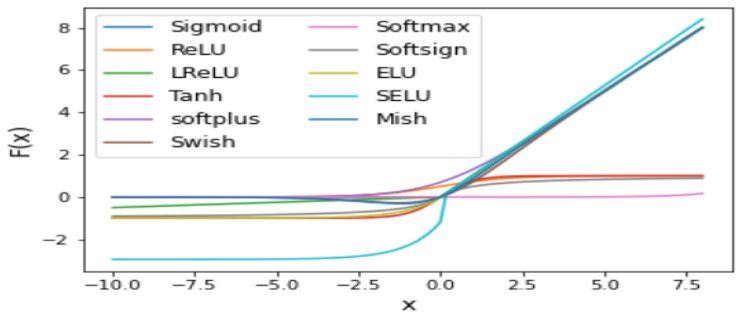
Pictorial Representation of Activation Function Responses.

This section presents the various types of activation functions and recent advances in existing activation functions employed in DL and ML applications. It highlights recent trends in utilizing the activation functions for deep learning-based vehicle detection, classification, and recognition. The most common activation functions used in Deep Learning architectures are shown in Figure 6.

Activation functions can be linear or non-linear, depending on the function they convey when monitoring the results of networks. This technique can be used for a variety of purposes. As an example of how it can be deployed, consider image classification, image segmentation, and machine translation, as well as finding objects such as cars and other vehicle types.

Most of the time, the affine transformation is used to conduct linear mapping from an input function to an output function in the hidden layers of the linear net architecture. The data *x* transformation is described in the following way, as shown in Equation (1).

(1) $$\begin{array}{c} f\left(x_{i}\right)=w^{T}+b_{i} \end{array}$$

Data input, weight, and biases are all represented by $x_{i}$, *w*, and $b_{i}$, respectively. Additional computation is then necessary to translate these linear outputs into non-linear outputs for the AF, notably to learn patterns in data from the mapping from Equation (2). These net architectures produce the following results:

(2) $$\begin{array}{c} Y=(w_{1}x_{1}+w_{2}x_{2}+w_{3}x_{3}+\ldots w_{d}x_{d}+b_{i}) \end{array}$$

Each layer’s output is fed into a subsequent layer until the final output is achieved, but, by default, they are linear. For each net, the anticipated output determines the type of AF deployed. Since the output is linear, non-linear results are not an issue. Transfer functions (TF) are applied to the outputs of linear net architectures to generate additional computation for the converted non-linear outputs. Mathematically, it is defined in Equation (3).

(3) $$\begin{array}{c} Y=\psi (w_{1}x_{1}+w_{2}x_{2}+w_{3}x_{3}+\ldots w_{d}x_{d}+b_{i}) \end{array}$$

where, ψ is the activation function coefficient.

The requirements for these activation functions include transforming the linear input signals and net architectures into non-linear output signals, which helps the learning of high-order polynomials outside one degree for deeper nets. Generally, the activation function maintains the dying gradients’ values, and the exploding gradient rises because of the derivative terms. These are achieved using various mathematical functions employed for network computing.

Table 6 presents a summary of the most popular activation functions used in DL applications, such as object detection, image classification, and object type recognition, and their positions in DL models, as shown in Table 7.

### 4.1. Loss Function in Deep Learning

Developing proper cost functions for CV-based tasks has been a long-standing research direction to improve the ability of the present schemes. Its primary purpose is to evaluate the difference between the actual value of the samples and the estimated value. The robustness and convergence of the recommended system mainly depends on the value of the cost function.

The CV society has witnessed progress in image classification and object detection in the recent years. Improvements to the framework design, of, for instance, single-step deep detectors and two-step deep detectors, have accelerated the state-of-the-art (STA) incredibly. Recently, several innovative approaches have been introduced in the cost function design and the loss-based training schemes for deep architectures. Liu et al. [85] proposed a powerful convergence simulation-driven evolutionary search approach (CSE–Autoloss) to speed up searches by regularizing the rationality of the loss candidates using two modules (convergence property verification (CPV) and model optimization simulation (MOS)).

The loss function consists of classification loss (Cls) and location loss (Lls). The deep two-step object detector algorithms equip a hybrid of both L1 loss and Cross-Entropy [86] for regression and Bbox classification. In contrast, the deep single-step object detector algorithms suffer from severe positive–negative instance imbalance, due to dense sampling of possible object locations. Lin et al. [37] proposed Focal Loss to solve the imbalance problem. However, optimizing object detectors with traditional detection approaches to loss functions may result in sub-optimal solutions due to limited connections with performance evaluation metrics. Therefore, Jiang et al. [87] predicted IOU during training, IOU loss series in IOU loss, bounded IOU loss, and generalized IOU loss. To directly optimize IOU between estimated and actual values, IOU loss and distance IOU loss are used. This work epitomizes the essence of developing practical loss functions toward better orientation with performance evaluation metrics for object detection tasks.

### 4.2. Classification Loss Functions in Deep Learning

This section explains the most common loss functions employed in Deep learning for classification tasks. Table 8 presents a summary of classification loss function formulae.

### 4.3. Location Loss Functions in Deep Learning

This section explains the most common loss functions employed in Deep learning for classification tasks. Table 9 presents a summary of Location loss function formulae.

Regression-based problems using loss functions have merit and limitations. Table 10 shows some of the pros and limitations of commonly used loss functions in regression-based problems.

## 5. Optimization Algorithms in Deep Learning

Optimization Algorithms (OAs) are vital approaches for updating DL/ML parameters and reducing the value of the loss function [88,89]. Understanding the principles of various OAs and their roles in hyperparameter tuning improve the performance of the DL/ML architectures. This is carried out by rapidly adjusting the weights and other parameters until the objective function convergence.

However, optimization provides a means to reduce the cost function for DL architectures. The aims of OA and DL are different. Substantially, optimization approaches explore the suitable architecture and reduce errors with less computational cost within the given dataset samples. Furthermore, several researchers have conducted experiments to solve the noticeable challenges using analytical and numerical solutions. The most common tricky optimization challenges in Deep Learning are vanishing gradient, local minima, and saddle points.

Back-Propagation (BP) is an approach to training nets. The approach repeats two process cycles, propagation and updating weights. Training errors from the output layer propagate to the other nodes backwards. Errors are utilized to compute the cost function’s gradient concerning the parameter in the net. Then, the gradient is fed to the optimization approach, which utilizes it to update the weights to diminish the cost function. Moreover, the gradient of the objective function is mainly dependent on the dataset samples utilized and the gradient descent approach employed [89].

The most well-known OAs, implemented in various methods to decrease the cost function and fasten the learning of the architectures, are the following: Gradient Descent (GD) [90], Stochastic Gradient Descent (SGD) [91], Nesterov Momentum (NM) [92], Adagrad [93], Adadelta [94], RMSProp [95], Adaptive Momentum (Adam) [96], and Adapg [88].

Gradient Descent (GD): GD is a well-known optimization algorithm [90]. It is a technique for decreasing an objective function F(δ) that is parametrized by an architecture’s parameters $\delta \varepsilon R^{d}$ by updating the parameters in the opposite direction of the gradient of the objective function F(δ). The learning rate, $\phi_{t}$, determines the size of the stages to reach a local minimum. Mathematically, it is defined in Equation (4).

(4) $$\begin{array}{c} \delta_{t+1}=\delta_{t}-\phi_{t}▽F\left(\delta_{t}\right) \end{array}$$

Hence, $\phi_{t}$ is the LR, and $▽F(\delta_{t})$ is the gradient of the cost function for the $t^{th}$ iterate.

Stochastic Gradient Descent (SGD): this updates the parameters ($\delta_{t}$) frequently, so the objective function is subject to wild swings, due to the SGD [91] algorithm’s rapid gradient computations and improvement. Nevertheless, a sluggish learning rate can improve SGD, resulting in a lengthy training period. In addition, the architecture’s speed is hampered by the frequent transfer of data between GPU memory and local memory. The mathematical process of the SGD algorithm is depicted in Equation (5).

(5) $$\begin{array}{c} \delta_{t+1}=\delta_{t}-\phi_{t}▽F_{i}\left(\delta_{t}\right) \end{array}$$

Hence, $F_{i}\left(\delta \right)≜l\left(y_{i},f_{\delta }\left(x_{i}\right)\right)$ at the $t^{th}$ iteration, randomly pick *i* and update the parameter.

Nesterov Momentum (NM): In this method, the gradient is calculated based on future positions of the parameters rather than the current positions of the parameters [92]. An increase in momentum does not indicate where the parameters end up. A mathematical representation of the NM algorithm can be found in Equation (6).

(6) $$\begin{array}{c} m_{t}=\beta_{t-1}+\left(1-\beta \right)▽F_{i}\left(\delta_{t}\right)\\ \delta_{t+1}=\delta_{t}-\alpha_{t}m_{t} \end{array}$$

where, β is the value of momentum (*m*) at the $t^{th}$ iteration.

Adagrad: The Adagrad is a well-known OA utilized in DL architectures [93]. It is an approach that selects the LR (ϕ) based on the situation. Since the gradient and LR values are inversely proportional, it is suitable for allocating with sparse data. Dean et al. [97] showed that Adagrad significantly enhanced the robustness of the SGD and they utilized it for training large-scale frameworks at Google to detect cats. It scales the LR (ϕ) for each parameter according to the history of the gradients for that parameter (δ), which is done by dividing the current gradient in the update rule by the sum of the past gradients. Mathematically, it is defined in Equation (7).

(7) $$\begin{array}{c} G_{t}=G_{t-1}+\Delta F{\left(\delta_{t}\right)}^{2}\\ \delta_{t+1}=\delta_{t}-\frac{\phi }{\sqrt{G_{t}}+\epsilon }\Delta F\left(\delta_{t}\right) \end{array}$$

where *G* is the sum of the past gradients and ϵ is a small value for numerical stability. However, the Adagrad approach has the disadvantage of treating all the past gradients equally and manually selecting global LR. It also uses exponentially weighted decay for the history gradients. It is suggested that an Adadelta algorithm solves these limitations.

Adadelta: The Adadelta optimization approach was derived from the Adagrad approach so as to improve the following limitations of the Adagrad [94]:

- The continual decay of $\phi_{s}$ throughout the training phase;
- The requirement for a manually selected global learning rate.

Thus, it combines the merits of the Adagrad and Momentum approaches. Mainly, it scales the LR based on the past gradient. Nevertheless, it only utilizes the latest time window instead of the whole history, as is the case for Adagrad. It also employs a component that serves a momentum term, which sums up historical updates. A mathematical representation of the Adadelta algorithm can be found in Equation (8)

(8) $$\begin{array}{c} E{\left[\Delta F\left(\delta \right)\right]}_{t}=\eta E{\left[\Delta F\left(\delta \right)\right]}_{t-1}+\left(1-\eta \right)\Delta F\left(\delta_{t}\right)\\ E{\left[\Delta F{\left(\delta \right)}^{2}\right]}_{t}=\eta E{\left[\Delta F{\left(\delta \right)}^{2}\right]}_{t-1}+\left(1-\eta \right)\Delta F{\left(\delta_{t}\right)}^{2}\\ \hat{\delta_{t}}=-\frac{\sqrt{E{\left[{\hat{\delta }}^{2}\right]}_{t-1}}+\epsilon }{\sqrt{E{\left[\Delta F{\left(\delta \right)}^{2}\right]}_{t}}+\epsilon }\Delta F\left(\delta_{t}\right)\\ E{\left[{\hat{\delta }}^{2}\right]}_{t}=\eta E{\left[{\left(\hat{\delta }\right)}^{2}\right]}_{t-1}+\left(1-\eta \right){\left(\hat{\delta }\right)}_{t}^{2}\\ \delta_{t+1}=\delta_{t}+{\left(\hat{\delta }\right)}_{t} \end{array}$$

where, η is weight decay and ϵ is a small value for numerical stability.

RMSProp: Tieleman et al. [95] proposed an RMSProp algorithm to solve the problem of the LR vanishing in the Adagrad approach. It makes use of the weight-decaying mean of previous gradients [98]. A mathematical representation of the Adagrad algorithm can be found in Equation (9).

(9) $$\begin{array}{c} E{\left[\Delta F{\left(\delta \right)}^{2}\right]}_{t}=\eta E{\left[\Delta F{\left(\delta \right)}^{2}\right]}_{t-1}+\left(1-\eta \right)\Delta F{\left(\delta_{t}\right)}^{2}\\ \delta_{t+1}=\delta_{t}-\frac{\phi }{\sqrt{E{\left[\Delta F{\left(\delta \right)}^{2}\right]}_{t}}+\epsilon }\Delta F\left(\delta_{t}\right) \end{array}$$

where, η is weight decay, ϵ is a small value for numerical stability, and ϕ is the learning rate.

Adaptive Momentum Estimation: The Adaptive Momentum Estimation [96] is an alternative method that calculates adaptive LRs for each parameter. Furthermore, it stores the exponential weighted-decaying mean of the historical squared gradients. It combines the RMSProp and momentum approaches with a bias correction mechanism. Adam’s update rule consists of the following steps, and, mathematically, it is defined in Equation (10).

(10) $$\begin{array}{c} m_{t}=\beta_{1}m_{t-1}+\left(1-\beta_{1}\right)\Delta F\left(\delta_{t}\right)\\ v_{t}=\beta_{2}v_{t-1}+\left(1-\beta_{2}\right)\Delta F{\left(\delta_{t}\right)}^{2}\\ \hat{m_{t}}=\frac{m_{t}}{1-\beta_{1}^{t}}\\ \hat{v_{t}}=\frac{v_{t}}{1-\beta_{2}^{t}}\\ \delta_{t+1}=\delta_{t}-\frac{\phi }{\sqrt{\hat{v_{t}}}+\epsilon }\hat{m_{t}} \end{array}$$

Hence, $\beta_{1}$ can be 0.9, $\beta_{2}$ can be 0.999, and ϵ is a small value for numerical stability. $m_{t}$ the mean gradient, $v_{t}$ is the uncentered variance of the gradients.

Adapg: The Adapg is also a new optimization algorithm, which combines both the Adadelta and Adam optimizers [88]. Mathematically, it is defined in Equation (11).

(11) $$\begin{array}{c} E{\left[\Delta F\left(\delta \right)\right]}_{t}=\eta E{\left[\Delta F\left(\delta \right)\right]}_{t-1}+\left(1-\eta \right)\Delta F\left(\delta_{t}\right)\\ E{\left[\Delta F{\left(\delta \right)}^{2}\right]}_{t}=\eta E{\left[\Delta F{\left(\delta \right)}^{2}\right]}_{t-1}+\left(1-\eta \right)\Delta F{\left(\delta_{t}\right)}^{2}\\ \hat{\delta_{t}}=-\frac{\sqrt{E{\left[{\hat{\delta }}^{2}\right]}_{t-1}}+\epsilon }{\sqrt{E{\left[\Delta F{\left(\delta \right)}^{2}\right]}_{t}}+\epsilon }E{\left[\Delta F\left(\delta \right)\right]}_{t}\\ E{\left[{\hat{\delta }}^{2}\right]}_{t}=\eta E{\left[{\left(\hat{\delta }\right)}^{2}\right]}_{t-1}+\left(1-\eta \right){\left(\hat{\delta }\right)}_{t}^{2}\\ \delta_{t+1}=\delta_{t}+{\left(\hat{\delta }\right)}_{t} \end{array}$$

where, η is a weight decay and ϵ is a small value for numerical stability.

The optimization algorithms have been widely utilized to reduce errors and accelerate architecture processing time with less computational cost by updating the parameters on the dataset samples. A comparison study [90] of optimization approaches for DL architectures using four publicly available datasets was conducted to investigate the efficiency of the approaches. The datasets were labeled as Faces in the Wild (LFW), MNIST, Kaggle Flowers, and CIFAR10 by pointing out their various attributes against SGD, NM, Adagrad, Adadelta, RMSProp, and Adam OAs. Zaheer et al. [99] conducted a study of OAs on training DL architectures involving the learning of the parameters to meet the loss function to reduce the loss during the training phase. They employed six methods using different datasets: MNIST, CIFAR10, FASHIONMNIST, and CIFAR100 on SGD, NM, Adagrad, Adadelta, RMSProp, and Adam approaches. They achieved the optimal training results for FASHIONMNIST 1.0 with RMSProp and Adam at 400 epochs, MNIST 1.0 with RMSProp and Adam at 200 epochs, CIFAR100 1.0 with RMSProp and Adam at 100 epochs, and CIFAR10 1.0 with RMSProp and Adam at 200 epochs. Their experimental results illustrated that the Adam optimizer performed outstandingly at the testing stage and RMSProp with Adam at the training step.

To summarize, RMSProp is Adagrad’s extension designed to alleviate the significantly reduced LR. It is identical to Adadelta, except that Adadelta utilizes the RMS of parameter updates in the numerator update rule. Finally, Adam summarizes bias correction and momentum to RMSProp. RMSProp, Adam, and Adadelta are similar approaches that outperform in related fashions. According to Zaheer et al. [99], its bias-correction aids Adam optimizer in outperforming RMSProp during testing and RMSProp with Adam during training. From various studies and papers, Adam might be the special optimization algorithm overall choice [100].

## 6. Application of DCNN for Vehicle Detection and Classification

This section discusses various difficulties and challenges in vehicle detection and classification, the application of DCNN, and a review of related works.

### 6.1. Difficulties and Challenges

This section discusses the difficulties and challenges of detecting, recognizing, and classifying vehicular objects.

Research communities have, for a long time, focused on the question, “What are the difficulties and challenges in vehicle object detection, classification, and recognition?” This question is not an easy one to answer, being a question that addresses other areas of object detection tasks, such as pedestrian detection and traffic sign detection and recognition. Various constraints, difficulties, and challenges arise in attempting to answer the question, depending on objectives and assignments [101]. However, the following are common challenges and difficulties frequently seen in appearance-based object detection and classification tasks: weather conditions, various camera viewpoints, vehicle size, vehicle color, vehicle inter-class variation; speed-up of classification and detection, correct vehicle localization, dense and occluded vehicle detection, and classification. Weather conditions, such as heavy fog, snowing, rain, snowstorms, dusty blasts, and low light conditions have a significant impact on detection accuracy and processing time. As a result of these conditions, visibility is inadequate for accurate detection of vehicles on the roads, resulting in traffic accidents. A clear view can be achieved by developing successful image enhancement techniques to gain good visuals. Providing clear images to detection systems can, thus, improve the performance of vehicle detection and tracking in intelligent visual surveillance systems and autonomous vehicle applications. Furthermore, by utilizing efficient image processing techniques [77], various vehicle detection approaches, such as Deep learning, ensemble learning, and other real-time-based vehicle detection using camera sensors, have grown in importance in autonomous vehicles due to their high detection accuracy, and have, thus, become significant in self-driving applications.

### 6.2. DL in Vehicle Detection

This section summarizes related works and their findings on vehicle detection using various DL approaches.

The rapid growth in digital image processing and computing systems has enabled the robust, accurate, and efficient employment of CV-based vehicle detection techniques. However, the framework efficiency mainly depends on the type of vehicles, illumination and light, size of vehicles, inter-class and intra-class variations, environment, and occlusion and blurred conditions. Considering these challenges and the difficulty of vehicle detection, directly utilizing generic detection networks is not an optimal solution. There may be some priors that can be used to improve vehicle detection. Table A1 summarizes related comparisons of real-time DL architectures from the literature review. The reason for the different reported results can be attributed to various factors: the type of loss function utilized, the different datasets used, various hyperparameters, the framework of the model, and the type of hardware used.

In the early stages of research, before the DL era, vehicle detection was mainly based on sliding windows, developed by Viola and Jones. Dense image grids were encoded by handcrafted features followed by a training classifier to explore and locate objects [102]. Haselhoff and Kummer [103] proposed a cascade of boosted classifier, Haar, and triangle features with a Kalman filter for vehicle detection, and achieved good performance in determining the vehicle’s position accurately. After the rapid growth of DL in image classification, vehicle detectors based on DL significantly outperformed traditional vehicle object detectors.

The current vehicle detection networks based on DL are extended from generic systems, such as YOLO, SPPNet, SSD, Faster RCNN, and Fast RCNN. Multi-scale learning methods have been used a lot in detecting vehicles because they can handle a lot of different sizes and scales.

Kim et al. [104] proposed a YOLOv3-based architecture that combined prediction layers using SPPNet to complement the detection accuracy for multi-scale variations in traffic surveillance data. Chen et al. [105] proposed an inception–SSD algorithm for small vehicle detection, which was found to be more suitable for vehicle detection on various aspect ratios and scales of default bounding boxes. They made predictions on the KITTI and UVD datasets. They developed a trade off between speed and vehicle detection accuracy, based on the SSD algorithm. To improve multi-scale detection, Zhao et al. [106] proposed the feature pyramid enhancement strategy (FPES) [44], based on semantic information, detailed features, and receptive fields [106]. Cascade detection and adaptive threshold acquisition approaches for the object detection module (ODM) stage were also presented to improve network accuracy.

Zhang et al. [107] developed an enhanced version of the RetinaNet technique to improve the representation of feature maps using octave convolution and to reduce gradient propagation in the extraction of multi-scale features by employing a weighted feature pyramid network (WFPN). Their approach effectively handled gradient propagation at various levels and low-resolution problems, but it was minor in performance. Unlike this approach, Wang et al. [108] proposed a focal loss-based RetinaNet algorithm, which was utilized to resolve issues of critical class imbalance in the standard one-step object detector, so as to improve performance.

Moreover, some algorithms focus on contextual information for multi-scale feature learning. Vehicle objects have a relationship with the surrounding context, namely, color, shadows, the structure of vehicles, and size and shape, which have become an effective means to improve detection performance. Hu et al. [73] proposed SINet, based on a scale-insensitive ConvNet for fast detection of vehicles with a significant variance in scales. They utilized context-aware RoI polling to handle the contextual information of the original structure of small objects. In addition, they proposed a multi-branch detection algorithm to reduce the intra-class distance features. Luo et al. [109] developed a state-of-the-art architecture that can be used to effectively detect multi-scale vehicle targets in traffic scenes. They increased the usage of the architecture in the following ways: NAS optimization and feature enrichment. There are several steps in this process. First, they implemented a Retinax-based image adaptive correction algorithm to improve image quality and minimize shadow and illumination effects. Then, they utilized a backbone model, NAS, for feature extraction in order to produce the best cross-layer connection for extracting multiple layers of features. Finally, they used object feature enrichment to integrate the multiple layers of features and contextual data.

Beyond designing robust or context-assisted object detectors, several studies have been conducted on various approaches. Nguyen et al. [81] proposed an improved system based on faster RCNN for fast vehicle detection. They replaced the NMS algorithm with the Soft-NMS algorithm to solve the problem of duplicate proposals, and a contextual-aware RoI pooling layer was adopted to adjust the proposals to a specified size without losing crucial contextual information. At the end of the MobileNet algorithm, the framework of depth-wise separable convolution is used to generate a classifier for each identified vehicle. Wang et al. [22] proposed an R-FCN algorithm equipped with deformable convolution and RoI pooling for vehicle detection. It has a better detection time and more precision. Wang et al. [35] conducted comparative studies on the most widely employed algorithms, Faster RCNN, RetinaNet, YOLOv3, RFCN, and SSD. They showed that RFCN is very powerful for generalizing real scenes and has outstanding detection on rainy days and at nighttime. Moreover, the SSD network also has good generalization ability and can detect most target vehicles in an environment with poor lighting conditions.

Arora et al. [110] recommended a fast RCNN architecture to detect vehicles under various environmental conditions. The proposed model obtained an average of recall, accuracy, and precision of 98.44%, 94.20%, and 90%, respectively. Charouh et al. [111] suggested a resource-efficient CNN-based model for detecting moving vehicles on large-scale datasets. Rajput et al. [112] proposed a toll management system, using Yolov3 architecture, for vehicle identification and classification. Amrouche and his colleagues proposed a Yolov4 architecture for a real-time vehicle detection and tracking system [113]. Wang et al. [114] introduced an integrated part-aware refinement network, which combines multi-scale training and component confidence generation strategies in vehicle detection. This system improves detection accuracy and time taken in detecting various vehicles on publicly available datasets.

Faris et al. [115] proposed a Yolo-v5 architecture vehicle detector using the techniques of transfer learning on publicly available datasets, namely, PKU, COCO, and DAWN. The experimental result showed that the proposed model achieved a state-of-the-art in the detection of various vehicles. Huang et al. [116] introduced an embedded system of Yolov4, K-means and TensorRT to detect the real-time target from UAV images. They achieved a confidence and miss detection rate of 89.6% and 3.8%, respectively. Furthermore, to balance the architecture’s detection accuracy and computational complexity, Qiu et al. [117] introduced a linear transform approach, increasing the detection accuracy and the detection frame using simple operations over the input image. However, the road and the various shapes and sizes of vehicles affect the system’s detection accuracy and detection frame in the detecting and recognizing scheme. Yolov7-RAR was proposed to minimize the miss detection of non-linear features and speed up the architecture in [118].

**Figure 7:**
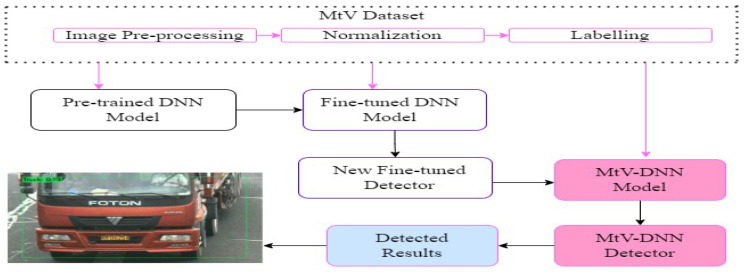
Vehicle Detection Process Based on Fine-tuned DNN.

To further improve detection accuracy, some researchers implemented an ensemble learning technique on pre-trained models. Mittal et al. [119] proposed an EnsembleNet model for vehicle detection and estimation of traffic density with a detection accuracy of 98%. Figure 7 is a sample block diagram of the vehicle detection process, using multi-type vehicle images, and based on fine-tuned DNN models.

### 6.3. DL in Vehicle Classification

This section summarizes related works and their findings in vehicle classification using various approaches.

Vehicle classification is a crucial part of the ITS and has several applications: intelligent parking systems, driver assistance, fleet management, maintenance systems, traffic flow statistics, automatic toll collection, accident analysis, investigation, and transportation system design and monitoring. With the rapid growth of image classification in recent years, much research has been done on computer vision-based vehicle classification using traditional object classifiers and CNN-based object classifiers, such as SVM, to train classification networks. However, the efficiency of the traditional approach is not robust due to unstable feature extraction from various changes, such as occlusion, blurring, illumination and lighting effects, environment, size and shape of vehicles, and diverse poses. Considering these problems in vehicle classification, directly employing the traditional approach is not an acceptable solution to classify vehicle categories/types in various conditions with a lower error rate. Further improvement in vehicle classifiers should be considered a core task.

Several kinds of research have been utilized in vehicle object classification tasks, namely vehicle type classification, vehicle damage type classification and detection, vehicle target classification and recognition, vehicle model, type, and manufacturer, color recognition, and vehicle counting. In recent years, diverse classifiers of model-based and vision-based approaches have been utilized. The model-based approaches recover the vehicle’s length, height, and width from various view images for vehicle classification. In contrast, the vision-based approaches extract appearance features from either vehicle side view, rear view, or front view images to classify vehicle types. Gupte et al. [120] proposed a non-rigid model-based approach to classifying vehicles by comparing the projection with the vehicle image to determine the class of the vehicle. Petrovic et al. [121] proposed a Sobel edge response type, direct normalized gradients, edge orientation, locally normalized gradients, and Harris approaches for integration to classify vehicle types. Psyllos et al. [122] proposed SIFT features to recognize the model, logo, and manufacturer of a vehicle. Peng et al. [123] introduced a system to designate a vehicle by vehicle front, color, type, and width for vehicle type classification. However, this approach utilizes handcrafted features and is difficult to visualize well enough. To handle the problems, Dong et al. [124] proposed a semi-supervised ConvNet algorithm for vehicle type classification on the BIT-vehicle dataset. They used sparse filtering to capture rich and discriminative information about vehicles. To improve the vehicle type classification of the model, Awang et al. [125] proposed an enhanced sparse-filtered ConvNet algorithm with a layer-skipping strategy (SF-ConvNetLS) to classify vehicle types. They employed three channels of SF–ConvNetLS as the feature extraction approach.

The DL outperformed conventional object classifiers after the rapid development of DL applications in image classification. The current vehicle object classifier based on DL has dramatically shifted from the model-based approach to the vision-based approach to improve classification accuracy and to resolve the challenges faced during real-time classification.

Several DL studies have been conducted to address classification problems since the excellent performance, exhibited by Krizhevsky et al. [24] in the ImageNet LSVTC [126] using DConvNets. Several DL studies have been conducted to address classification problems. Szegedey et al. [28] introduced a novel DNN using Inception networks that maximize the depth of architectures without increasing the number of parameters. Simonyan and Zisserman [30] demonstrated that 3×3 receptive fields in the first conv layers were more effective than 11×11 receptive fields with stride four or 7×7 with a stride of 2, which improved the performance on ILSVRC.

Manugmai and Nuthong [127] proposed a DL-based vehicle classification approach to classify vehicle type and color. They showed that the ConvNet architecture outperformed the conventional machine learning approaches in classification. Wang et al. [128] proposed AVC using center-strengthened ConvNet to extract more features from a central image by ROI pooling, based on the VGG model joined with the ROI pooling layer to obtain elaborate feature maps. Awang and Azmi [129] presented a ConvNet architecture with a skipping strategy model to classify vehicles with identical sizes of different object classes, and Jahan et al. [130] proposed real-time vehicle classification using ConvNet. They used two ways to find features and classify different types of vehicles.

Lee and Chung [131] proposed a DL-based vehicle classification using an ensemble of *K* local experts and global networks. They used multi-crop testing, network training of *k* local experts, and global networks with an ensemble of AlexNet [126], ResNet [29], and GoogleNet [28] to classify various vehicles.They achieved outstanding performance on the MIT–CCD classification challenges. In order to improve the mean precision of the models, Liu et al. [132] proposed a two-step approach of DA and an ensemble of ConvNet algorithms to solve the imbalance dataset problem in calibrating with hyperparameter optimization of parameters. They showed that the ensemble technique with DA improved the precision. Liu et al. [80] presented a semi-supervised network motivated by a combination of various DNNs with DA techniques based on GAN. It includes several steps to improve classification accuracy on the MIO–TCD dataset.

Furthermore, Jagannathan et al. [133] proposed a GMM and ensemble DL approach to detect and classify various moving vehicles on both the BIT-vehicle dataset and the MIO-TCD dataset. They utilized adaptive histogram equalization and GMM to improve image quality, and a steerable pyramid transform and Weber local descriptor (WLD) were used to extract feature vectors. Then, the extracted feature vectors were fed into the ensemble Dl approach for the vehicle classification task. They showed that the proposed model outperformed the benchmark models on both datasets.

Table A2 summarizes the comparison of DL-based vehicle classification architectures from the literature review. The type of loss function used, the different datasets used, different hyperparameters, the framework of the model, and the type of hardware used all lead to different results.

## 7. Future Directions

Despite the rapid growth and promising object detection and classification processing in DL applications, there are still several open issues for future work.

Various methods for detecting and classifying small vehicles in publicly available datasets have been developed. To enhance the classification and localization accuracy of small vehicle objects under several occlusions, inter-class variation, intra-class variation, illumination, light, environment, etc. it is necessary to modify the model architecture in the following aspects:

Multi-task joint optimization and Multi-model information combination: Due to the relationship between several tasks in vehicle object classification and detection, Multi-task joint optimization has been studied by several researchers, such as the following: in person re-identification [134], human action grouping and recognition [135], dangerous object detection [134], fast object detection [136], multi-task vehicle recognition and tracking [137], multi-task vehicle pose estimation [138]. Moreover, several approaches have been integrated to improve the performance of the architectures.

Scale and size alteration: Objects typically appear in a variety of scales and sizes, which is more noticeable in small objects. For scale- or size-variant objects, multi-scale object classifiers and detectors are required to maximize the robustness to scale and size changes. Powerful backbone algorithms, such as ResNet, Inception, MobileNet, and AlexNet, can be utilized for scale-/size-invariant detection and classification tasks. FPN generates multi-scale feature maps and GAN-based narrow representation variations between small and vast objects with lower computational complexity for the multi-scale detectors and classifiers. The network offers insights into producing a meaningful feature pyramid for scale-adaptive detectors. It is necessary to integrate cascade architecture and scale distribution estimation to identify objects adaptively.

Spatial Correlations and Contextual Modeling: Spatial distribution plays an essential role in object detection and image classification. Therefore, region proposal generation and grid regression are employed to get probable object locations. However, the corrections between several proposals and object classes are disregarded. In addition, the global structure information is uncontrolled by position-sensitive score maps in RFCN. To solve these problems, use of various techniques, such as sequential reasoning tasks and subset selection, in a collaborative way is advocated.

Cascade Architecture: In the cascade network, a cascade of detectors is built in several phases. However, the existing cascade architectures are made greedy, where previous phases in cascades are fixed when training a new phase. So, the optimization of different ConvNets cannot be accomplished, which makes the need for end-to-end optimization for the ConvNet cascade architecture even more important.

Weakly supervised and Unsupervised Learning: Practically, it is inefficient and labor-intensive to label a large volume of bounding boxes manually. To address this issue, different architectures can be combined to perform exceptionally well by utilizing image-level supervision to assign object classes to match object regions and object boundaries. This technique leads to improved detection flexibility and minimized labor costs.

Model Optimization: A technique of model optimization in DL applications and schemes is essential to balance accuracy, speed, and memory, by choosing an optimal detector and classifier.

Detection or Classification in Videos: Real-time object classification and detection in videos is a significant issue for video surveillance and autonomous driving. Conventional object classifiers or detectors are usually designed for image-wise detection and classification, while simply ignoring the correlations between video frames. An essential direction of research is to enhance detection or classification performance by searching for spatial and temporal correlations.

Lightweight Classification or Detection: The lightweight architectures have been greatly compromised by classification errors developing in models. There is still a shortage of detection accuracy. While great efforts have been made in recent years, the speed of detection and of classification speed are not yet balanced.

## 8. Conclusions

In this paper, a comprehensive survey of some of the significant growth, successes, and demerits associated with applying DL techniques in vehicle (object) detection and classification is presented. To prove the efficiency of applying DL techniques in vehicle (object) detection and classification, benchmark datasets, loss functions, activation functions, and various experiments and studies recently implemented and completed in vehicle detection and classification are reviewed. Detailed analysis of deep learning techniques and reviews of some significant detection and classification applications in vehicle detection and classification, in-depth analysis of their challenges and promising technical improvements in recent years are addressed. Finally, we suggest many future directions in thoroughly understanding the object detection and classification landscape. This survey is also meaningful for the growth of Nets and related learning frameworks, which offer valuable insights and guidelines for future progress.

## Figures and Tables

**Table 1:** Summary of the Two-step Algorithms in Object Detection and Classification Applications.

Algorithms	Advantage	Disadvantage
RCNN [23]	Utilizes selective search approach to produce regions. Extracts 2000 regions from each image than the standard CNN algorithm.	High computational time. Slow speed because of using several networks for generating predictions. Difficult to detect small-scale objects
Fast RCNN [26]	Each image is passed only once to the CNN algorithm, and feature maps are extracted. Selective search approach is employed on these maps to produce predictions.	Requires a high volume of the real-time dataset. High computation time.
Faster RCNN [27]	Replaces the selective search approach with RPN algorithm, which makes the algorithm much faster	Requires several passes using a single image to extract all the object classes. The performance of the algorithms depends on how the preceding schemes have performed.
RFCN [31]	Uses position-sensitivity score maps to solve the position sensitivity problem of object classification and detection. Has less computational time compared to the rest of the algorithms, due to its property of sharing every convolutional layer.	R-FCN has a competitive mAP but it is lower than that of Faster R-CNN.

**Table 2:** Summary of the Single-step Algorithms in Object Detection and Classification Applications.

Networks	Advantage	Disadvantage
SSD	Simple neural network. low computational expensive	Low detection accuracy in complex scenarios.
RetinaNet	Enhanced detection precision on small objects. suitable for class imbalance training process	Requires real-time detection.
YOLOv1	Fast compared to the two-step object detectors. global trainable module stops optimization. offers higher generalization when evaluating another dataset.	Poor performance for a set of small object classes, due to its grid set-up. high localization error.
YOLOv2	It dramatically enhances the speed and accuracy of object detection. It is easy to detect objects with grids and boundaries prediction, and also it helps in predicting tiny objects or objects that are very far in the image	Complex Training
YOLOv3	Fast, robust predictions of objects in real-time. computational inexpensive.	Worst to detect medium and large objects.
YOLOv4	Excellent detection accuracy. better training optimization	Poor small target detection accuracy.
YOLOv5	Outstanding detection/recognition accuracy. low false detection rate. works efficiently. low computational cost. easily to set up.	Has both global maxima and local minimal.

**Table 3:** The summary of Performances of the Various Algorithms Employed in Object Detection.

Networks	Backbone	Dataset	Image Size	AP@0.5	AP@0.5 to 0.95	FPs
RCNN	AlexNet	PASCAL VOC 12	224	-	58.50	0.02
Fast RCNN	VGG-16	PASCAL VOC 12	variable	-	65.70	0.43
Faster RCNN	VGG-16	PASCAL VOC 12	600	-	67.00	5
R-FCN	ResNet-101	COCO 12	600	31.50	53.20	3
RetinaNet	ResNet-101-FPN	COCO 12	400	31.90	49.50	12
SSD	VGG-16	COCO 12	300	23.20	41.20	46
YOLOv1	GoogleNet	PASCAL VOC 12	448	-	57.90	45
YOLOv2	DarkNet-19	COCO 12	352	21.60	44.00	81
YOLOv3	DarkNet-53	COCO 12	320	28.20	51.50	45
YOLOv4	CSPDarkNet-53	COCO 12	512	43.00	64.90	31

**Table 4:** Existing Works’ Performance outcomes.

References	Approach	Dataset	Evaluation Metrics
Zuraim et al. [78]	Yolov4.DeepSORT.	Own dataset.	82.08% of average precision.
Xu et al. [79]	Modified YOLOv3 classifier.	VEDAI dataset.	91.72% of average precision.
Liu et al. [80]	BFEN + SLPN + PNW.	DETRAC benchmark dataset.	88.71% of mAP.
Nguyen et al. [81]	Soft NMS algorithm. Faster RCNN classifier.	KITTI dataset. LSVH dataset.	83.92% average precision in the KITTI dataset. 64.72% average precision in the LSVH dataset.
Dai et al. [82]	Faster RCNN + SSD classifier.	KITTI dataset. PASCAL2007 car dataset.	85.22% average precision in the KITTI dataset. 64.83% average precision in the PASCAL2007 car dataset.
Nguyen et al. [83]	Faster RCNN with FPN backbone.	KITTI dataset. PASCAL2007 car dataset.	88.95% average precision in the KITTI dataset. 78.84% average precision in the PASCAL2007 car dataset.
Fan et al. [84]	Faster RCNN classifier.	KITTI dataset.	83.36% verage precision.

**Table 5:** Summary of Various Performance Evaluation Metrics.

Evaluation Metrics	Mathematical Formulae
Precision	$P=\frac{TP}{TP+FP}$
Recall	$R=\frac{TP}{TP+FN}$
Frame Per Second	$FPS\left[n\right]=\frac{n*FPS[n-1]}{FPS[n-1]*FPS[n-1]+n-1}$
Intersection over Union (IoU)	$J\left(Bbox_{p},Bbox_{g}\right)=\frac{area(Bbox_{p}⋂Bbox_{g})}{area(Bbox_{p}⋃Bbox_{g})}$
Average mean Precision	$mAP=\frac{1}{n}\sum_{i=1}^{n}AP_{i}$
Average Precision	$AvP=\sum_{n}\left(Re_{n+1}-Re_{n}\right)\max_{Re:\hat{R}e\geq Re_{n+1}}P\left(\hat{R}e\right)$
True Positive Rate	$TPR=\frac{TP}{TP+TN}$
False Positive Rate	$FPR=\frac{FP}{FP+TN}$
Accuracy	$Accuracy=\frac{TN+TP}{TP+FP+TN+FN}$
F1-Score	F1-$Score=\frac{2\times R\times P}{R+P}$
Area Under Curve	$AUC=\frac{1}{2}-\frac{FPR}{2}+\frac{TPR}{2}$

**Table 6:** Summary of the Activation Functions in DL Applications.

Functions	Formula	Advantage	Disadvantage
Sigmoid	$f\left(x\right)=\frac{1}{e^{-x}+1}$	Suitable for light Networks. Used in feedforward NNs. Bounded and differentiable actual function.	Dramatically declines gradients during back-propagation. Has the nature of gradient saturation. Slow convergence and non-zero centered output lead the gradient updates to propagate in various directions.
Tanh	$f\left(x\right)=\frac{e^{x}-e^{-x}}{e^{-x}+e^{x}}$	It presents outstanding training performance for MLP NNs. Generates zero centered output to assist the bac-kpropagation process.	It generates dead neurons during computation. High degree computational complexity.
ReLU	$\begin{array}{c} \frac{x}{1+e^{-x}},\;\mathrm{if}\;x\;>\;0\\ \frac{e^{-x}-1}{1+e^{-x}},\;\mathrm{if}\;x\;<\;0 \end{array}$	Faster learning activation compared to others. Most successful and widely employed function. Presents outstanding performance and generalization in DL architectures compared to sigmoid and Tanh functions. Simple to optimize. No gradient saturation problems. Low computational cost.	It has the nature of over-fit compared to a sigmoid function. Insubstantial during the training process and leads to some of the gradients dying. It is not a zero-centered function.
ELU	$\begin{array}{l} f_{ELiU}\left(x\right)=x,\;\mathrm{if}\;x\;>\;0\\ \Gamma e^{x}-1,\;\mathrm{if}\;x\;\leq \;0 \end{array}$	It can solve the problem of gradient vanishing using identity values. Ability to learn characteristics of DL systems improves. Can minimize the computational complexity of using the mean unit action function.	A high degree of computational complexity.
Softmax	$f\left(x\right)=\frac{e^{x_{i}}}{\sum_{j}e^{x_{i}}}$	It is used for multivariate classification tasks.	Not suitable for binary classification problems.
Softplus	$f\left(x\right)=\log\left(1+e^{x}\right)$	It has smoothing and non-zero gradient properties to improve stabilization and performance of DL with fewer epochs to convergence during the training process. It can handle the vanishing gradient problem.	A high degree of Computational complexity.
Swish	$f\left(x\right)=\frac{x}{e^{-x}+1}$	Uses automatic search approaches to compute the function. Presents outstanding optimization and generalization outcomes. Does not suffer from problems of gradient vanishing. It requires simple scalar inputs.	A high Computation complexity.
ELiSq	$\begin{array}{c} \frac{x}{1+e^{-x}},\mathrm{if}\;x\;>\;0\\ \frac{e^{-x}-1}{1+e^{-x}},\mathrm{if}\;x\;<\;0 \end{array}$	It presents excellent optimization and generalization outcomes. Does not suffer from problems of gradient vanishing. Requires simple scalar inputs. It reduces the problem of the gradient vanishing to improve information flow.	
Maxout	$f\left(x\right)=max\left(w_{1}^{T}x+b_{i},\cdot \cdot ,\cdot \cdot ,w_{n}^{T}x+b_{i}\right)$	Easily to generalize.	A high computational complexity.

**Table 7:** Types and Positions of Activation Functions in DL Models.

Models	Hidden Layers	Output Layers
SeNet	ReLU	Sigmoid
ReseNeXt	ReLU	Softmax
AlexNet	ReLU.	Softmax
DenseNet	ReLU.	Softmax
GoogleNet	ReLU.	Softmax
EfficienNet	ReLU.	Softmax
MobileNet	ReLU.	Softmax
ResNet	ReLU.	Softmax
ImageNet	ReLU.	Softmax
SqueezNet	ReLU.	Softmax
VGGNet	ReLU.	Softmax
Inception	ReLU.	Softmax

**Table 8:** Summary of Classification Loss Functions in Deep Learning.

Loss Functions	Mathematical Formula
Hinge Loss	L(z)=max(0,1−t.Z)z=w.X+b
Squared Hinge Loss	$L\left(Q,\hat{Q}\right)=\sum_{j\neq i}^{n}\left(max{\left(0,1-Q_{i}.\hat{Q_{i}}\right)}^{2}\right)$
Kullback–Leibler Divergence	$\begin{array}{l} D_{KL}\left(E||B\right)=\sum_{i}E_{\left(i\right)}log\frac{E_{\left(i\right)}}{B_{\left(i\right)}}\\ =\sum_{i}E_{\left(i\right)}(logE_{\left(i\right)}-logB_{\left(i\right)})\\ =\sum_{i}E_{\left(i\right)}(logE_{\left(i\right)}-\sum_{i}E_{\left(i\right)}logB_{\left(i\right)} \end{array}$
Cross Entropy Loss	$L\left(P,\gamma \right)=\sum_{i=0}^{n}\gamma_{i}\log P_{i}$

**Table 9:** Summary of Location Loss Functions in Deep Learning.

Location Loss Functions	Mathematical Formula
Absolute Loss	L(Y,f(X))=|y−f(x)|
Sum of Absolute Differences	$L\left(Y,f\left(X\right)\right)=\sum_{i=1}^{n}\left|y_{i}-f\left(x_{i}\right)\right|$
Mean Absolute Error	$L\left(Y,f\left(X\right)\right)=\frac{1}{n}\sum_{i=1}^{n}\left|y_{i}-f\left(x_{i}\right)\right|$
Mean Square Error	$L\left(Y,f\left(X\right)\right)=\frac{1}{n}\sum_{i=1}^{n}{\left|x_{i}-y_{i}\right|}^{2}$
Huber Loss	$\frac{1}{2}{\left(y-f\left(x\right)\right)}^{2},\mathrm{for}\;|y-f(x)|\leq \lambda$ *λ*|*y* − *f*(*x*)| − $\frac{1}{2}$ *λ*^2^, otherwise

**Table 10:** Summary of the Loss Functions in Regression-based Problems.

Loss Functions	Advantage	Disadvantage
Mean Square Error Loss	The GD has only global minima. No local minima. penalizes the network architecture for making large mistakes.	Not robust if the samples consist of outliers.
Mean Absolute Error Loss	More robust compared to MSE.	High computational cost. Has a local minima. large global for small loss
Huber Loss	Outliers are handled wisely. No local minima. It is differential at zero.	Requires extra hyperparameter optimization techniques.

## Data Availability

Not applicable.

## Abbreviations

The following abbreviations are used in this manuscript:
  Adam	Adaptive Momentum
  AI	Artificial Intelligence
  BP	Back-propagation
  CV	Computer-vision
  DCNNs	Deep Convolutional Neural Networks
  DL	Deep Learning
  DNNs	Deep Neural Networks
  EL	Ensemble Learning
  FC	Fully Connected
  GD	Gradient Descent
  GPUs	Graphic Processing Units
  HOG	Histogram of Oriented Gradient
  ITS	Intelligence Transportation System
  LBP	Local Binary Pattern
  LR	Learning Rate
  ML	Machine Learning
  OAs	Optimization Algorithms
  RCNNs	Regional Convolutional Neural Networks
  SGD	Stochastic Gradient Descent
  TL	Transfer Learning

## Appendix A

### Appendix A.1

**Table A1:** Summary of Various Algorithms and Datasets utilized in Vehicle Detection.

Reference	Dataset Used	Network	Findings
Sang et al. [139]	BIT-vehicle dataset. Training 7880. Validation 1970. Testing (CompCar dataset) 800	YOLOv2. Model-Comp YOLOv2-vehicle	YOLOv2-vehicle has higher precision and average IOU than YOLOv2 and model-Comp. Model-Comp has a higher average IOU than YOLOv2.
Xu et al. [79]	COCO dataset	YOLOv3. improved YOLOv3. Faster RER-CNN. Modified YOLOv3	The modified YOLOv3 has higher average precision than improved YOLOv3, YOLOv3, and Faster RER-CNN.
Liu et al. [80]	DETRAC dataset	Faster RCNN. EB., BFEN. BFEN + 2FC. BFEN + SLPN. BFEN + SLPN + PNW.	The BFEN + SLPN + PNW has higher than Faster RCNN, EB, BFEN, BFEN + 2FC, and BFEN +SLPN.
Mansour et al. [140]	From JF-2 and WORLD-VIEW satellites	Faster RCNN + Inceptionv2. SSD + Inceptionv2.	Faster RCNN with Inceptionv2 has higher mAP than SSD with Inceptionv2 but has a higher operation time than SSD with Inceptionv2.
Sowmya et al. [141]	COCO test set PASCAL VOC 07 test set	ResNet101, VGG16 RCNN(Alex) RCNN (VGG16) SPPNet YOLOv4 + DA + TL.	YOLOv4 + DA + TL has higher mAP than ResNet101, VGG16, RCNN(Alex), RCNN(VGG16), and SPPNet.
Nguyen [81]	KITTI test set LSVH test dataset.	Faster RCNN SSD MSCNN YOLO YOLOv2 improved Faster RCNN.	The improved Faster RCNN algorithm has higher AP than the original Faster RCNN, SSD, MSCNN, YOLO, and YOLOv2 on the KITTI test set. MS-CNN has higher AP than Improved Faster RCNN on the LSVH test set.
Wang et al. [142]	DETRACT dataset.	Faster RCNN PN + FTN + Fusion PN + FTN + Concant PN + FTN + Fusion + Concant.	PN + FTN + Fusion + Concant has higher overall mAP than Faster RCNN, PN + FTN + Fusion.
Nguyen [83]	KITTI benchmark. PASCAL VOC 07.	DPM, Fast RCNN Faster RCNN, YOLOv2 Faster RCNN with FPN backbone MS-CNN improved Faster RCNN, SINet Multitask CNN Faster RCNN with FPN + Improving RPN + multilayer enhancement module + adaptive RoI pooling.	Faster RCNN with FPN + Improving RPN + multilayer enhancement module + adaptive RoI pooling has higher AP than DPM, Fast RCNN, Faster RCNN, YOLOv2, Faster RCNN with SPP, Improved Faster RCNN, SINet and Multitask CNN on both datasets.
Kim et al. [143]	DETRACT test set.	DPM, RCNN, ACF, Faster RCNN2 SA-FRCNN NANO CompACT, MSVD-SPP.	The MSVD-SPP has higher mAP than DPM, RCNN, ACF, Faster RCNN2, SA-FRCNN, NANO, and CompACT.
Wang et al. [144]	KITTI test set.	YOLOv2, tiny YOLOv2, tiny YOLOv3. SPPNet-YOLOv3	SPPNet-YOLOv3 has higher mean average precision than YOLOv2, Tiny YOLOv2, and Tiny YOLOv3.

### Appendix A.2

**Table A2:** Summary of Various Algorithms and Datasets utilized in Vehicle Classification.

Reference	Dataset Used	Network	Findings
Manugmai and Nuthong. [127]	Own dataset. Training = 686. Testing = 228	CNN. Decision tree Random forest DNN (Densely)	The CNN architecture has higher classification accuracy than DNN (densely), Decision tree, and random forest.
Wang et al. [128]	Caltech256 dataset	VGG-s. VGG-verydeep-16. CS-CNN.	The CS-CNN has higher accuracy than VGG-s and VGG-very deep-16.
Jahan et al. [130]	own dataset. Training = 2240. Testing = 560	YOLOv3. improved YOLOv3. Faster RER-CNN. Modified YOLOv3	The modified YOLOv3 has higher average precision than improved YOLOv3, YOLOv3, and Faster RER-CNN.
Lee and Chung [131]	MIO-CTD dataset	AlexNet ResNet18 GoogleNet ensemble learning (AlexNet + ResNet18 + GoogleNet).	The ensemble model of AlexNet, ResNet18, and GoogleNet have lower error rates than the benchmark models.
Liu et al. [132]	MIO-CTD dataset	ResNet50 ResNet50-BS ResNet101 ResNet101-BS ResNet152 ResNet152-BS DCEM DCEM-BS.	The DCEM-BS has higher precision than ResNet50, ResNet50-BS, ResNet101, ResNet101-BS, ResNet152, ResNet152-BS and DCEM. ResNet152-BS has higher mean recall than ResNet50, ResNet50-BS, ResNet101, ResNet101-BS, ResNet152, and DCEM.
Liu et al. [80]	MIO-CTD dataset	ResNet50 ResNet101 ResNet152 Inceptionv4 Inceptionv3 GEM-OE. GEM-AP.	GEM-AP has higher precision than baseline networks and GEM-OE. GEM-OE has higher precision than baseline architectures.
Jagannathan et al. [133]	MIO-CTD dataset BIT-vehicle dataset	GAN-based deep ensemble approach tiny YOLO with SVM semi-supervised CNN PCN with Softmax TC-SF-CNNLS.	Ensemble deep learning approach has higher recall than tiny YOLO with SVM, semi-supervised CNN, PCN with Softmax, and TC-SF-CNNLS. TC-SF-CNNLS has higher recall than tiny YOLO with SVM, semi-supervised CNN, and PCN with Softmax.
